# Special issue: The harms and benefits of cannabis and cannabinoids in women and men and effects on their progeny

**DOI:** 10.1016/j.dadr.2026.100409

**Published:** 2026-01-05

**Authors:** Rachel A. Rabin

**Affiliations:** a Department of Psychiatry, McGill University, Montreal, Canada; b The Douglas Mental Health University Institute, Verdun, Canada

With approximately 4.4 % of the global population using cannabis (United Nations Office on Drugs and Crime, 2024), a growing body of literature has examined both the potential harms and therapeutic effects of cannabis and cannabinoids in women and men, as well as impacts on offspring. Importantly, cannabis use during pregnancy has increased across several high-income countries, including the United States, Canada, Australia, and the United Kingdom (Singh et al., 2020). Reported prevalence estimates of prenatal cannabis use range from 2 % to 36 %, reflecting differences in study populations (e.g., mother’s age, socioeconomic level), definitions of use, and assessment methodologies (Beatty et al., 2012, Singh et al., 2020). This widespread rise in use may be due to changes in cannabis’ legal and regulatory frameworks which have contributed to greater availability, social acceptance, and perceived safety of cannabis (Chang et al., 2019, Volkow et al., 2019, Wilkinson et al., 2016), despite escalating concentrations of delta-9-tetradhyrocannabinol (ElSohly et al., 2021), its primary psychoactive and addictive constituent.

The highest frequency of cannabis use during pregnancy is reported in the first trimester and generally declines across gestation (Alshaarawy and Anthony, 2019, Young-Wolff et al., 2021). Many pregnant women report using cannabis to self-medicate pregnancy-related symptoms, such as pain, nausea, stress, and sleep and appetite disturbances (Constantino-Pettit et al., 2024, Gunn et al., 2024, Vanstone et al., 2021), often under the assumption that it is a safer alternative compared to conventional pharmacotherapies (Zaugg et al., 2024). However, emerging evidence suggest that potential adverse effects on fetal development may outweigh maternal therapeutic benefits.

Cannabinoids are highly lipophilic and readily cross the placenta, accumulating in fetal plasma in high concentrations (Thompson et al., 2019). Once in circulation, cannabinoids engage the endocannabinoid system both within placental tissue and the developing central nervous system (Harkany et al., 2007). Disruption of tightly regulated endocannabinoid signaling during this period has been shown to interfere with key neurodevelopmental processes (Lo et al., 2024). Although the nature and extent of these effects are only beginning to emerge, national guidelines recommend that pregnant individuals abstain from cannabis (Braillon and Bewley, 2018).

The contributions to this special issue highlight recent efforts to characterize the potential risks and therapeutic effects of cannabis and cannabinoids in women/females and men/males (Ganesh et al., 2024, Lin et al., 2024, Mills et al., 2025, Varas et al., 2025), as well as their impacts on fetal, neonatal, and cognitive, behavioral, and mental health outcomes in offspring (Argote et al., 2025, Azubuike et al., 2025, Cupo et al., 2024, Przy et al., 2025, Rouzer et al., 2025). These studies also examine how pregnant individuals and clinicians obtain, interpret, and communicate information regarding cannabis use, how these processes influence decision-making and prenatal care practices, and the role of stigma in shaping these interactions (Denson et al., 2025, Howe et al., 2025, Mian et al., 2025, Skelton et al., 2025, Sokol et al., 2025). Understanding the short- and long-term consequences of prenatal exposure to cannabis and cannabinoids on offspring is essential for equipping the public, clinicians, and policymakers with the evidence needed to make informed decisions regarding cannabis use.

Evidence from human and preclinical studies suggest that repeated cannabinoid exposure and use in the context of mental health symptoms may increase vulnerability to cannabis use disorder (CUD). Mills et al. (2025) examined the prevalence of, and factors associated with, CUD among adults using prescribed or illicit medical cannabis. Results indicated that high frequency cannabis use and co-occurring mental health conditions were key risk factors in the development of CUD. This aligns with findings from Lin et al. (2024), who reported on real-world prescribing data for purified cannabidiol (CBD; Epidiolex®) showing that individuals frequently seek cannabinoid-based therapies to attenuate mental health symptoms, such as anxiety and mood disorders, that lack formal FDA approval (Lin et al., 2024). Complementing these studies, preclinical work by Varas et al. (2025) showed that repeated cannabis exposure induces tolerance to its effects on exploratory behavior, a behavioral adaptation that may promote dose escalation and increase risk for CUD. Together, these findings highlight potential unintended risks of widespread cannabinoid use, particularly when used to manage mental health symptoms, which is common among pregnant individuals (Gunn et al., 2024).

Cannabis has gained attention as a pain management modality (Elikkottil et al., 2009), with emerging interest in its possible opioid-sparing properties (Nielsen et al., 2022). In a qualitative study of people who inject drugs, Ganesh et al. (2024) observed that some individuals used cannabis following opioid cessation to manage withdrawal-related symptoms, including anxiety and cravings (Ganesh et al., 2024). In this context, cannabis may be perceived by some individuals as a harm-reduction strategy for opioid use. However, cannabis use has also been associated with current problematic opioid use and an increased likelihood of future opioid misuse (Fiellin et al., 2013). Accordingly, careful clinical monitoring and guidance are essential when considering cannabis use in populations with opioid use or opioid use disorder.

Across our article collection, four review papers concluded that prenatal and early-life exposure to cannabis and cannabinoids is associated with neurodevelopmental consequences in offspring, while also indicating substantial methodological and translational gaps (Argote et al., 2025, Azubuike et al., 2025, Cupo et al., 2024, Przy et al., 2025). Cupo et al. (2024) conducted a narrative review integrating human and preclinical evidence, highlighting the complementary strengths and limitations of each approach and emphasizing the need for improved exposure characterization, closer alignment between laboratory models and real-world patterns of use, and longitudinal follow-up across development (Cupo et al., 2024). Extending this mechanistic perspective, Przy et al. (2025) systematically reviewed preclinical studies examining the effects of perinatal cannabinoid exposure on learning and memory outcomes. Their findings identified alterations in hippocampal synaptic plasticity as a neurobiological mechanism underlying cognitive dysfunction, while also noting heterogeneity in dose, timing, and cannabinoid composition (Przy et al., 2025). Azubuike et al. (2025) synthesized human evidence on neurodevelopmental and functional outcomes following in utero and early childhood (“second-hand”) cannabis exposure. The most consistent associations that emerged were between prenatal cannabis exposure and attention-deficit/hyperactivity disorder; associations with other outcomes, including depression, anxiety, and autism spectrum disorder, were less consistently supported (Azubuike et al., 2025). Finally, Argote et al. (2025) examined prenatal cannabis use in the context of tobacco co-use, given that these two substances are often used in combination both in the general population (Rabin and George, 2015) and among pregnant individuals (Coleman-Cowger et al., 2017). Findings revealed that co-exposure may exert negative additive or interactive effects on offspring outcomes, particularly with respect to neonatal, behavioral and physiological outcomes (Argote et al., 2025).

Co-use of cannabis with substances other than tobacco is also common. Notably, co-use with alcohol is highly prevalent among young adults of reproductive age (Subbaraman and Kerr, 2015) and therefore represents another common and clinically relevant form of prenatal co-exposure. Accordingly, Rouzer et al. (2025) investigated the effects of prenatal co-exposure to alcohol and synthetic cannabinoids in mice. Prenatal co-exposure was associated with increased offspring mortality relative to controls and resulted in elevated hyperactivity and anxiety-like behaviors in young adulthood compared with both single-substance exposure and control groups (Rouzer et al., 2025).

Despite growing evidence of adverse outcomes associated with prenatal cannabis exposure, perceptions of harm remain mixed. Denson et al. (2025) reported that many women perceive cannabis as natural, safe, and effective for managing pregnancy-related symptoms (e.g., nausea, stress, sleep), often underestimating fetal risks (Denson et al., 2025). Although most participants were aware of potential harms, these risks were frequently perceived as lacking credibility, and participants expressed a desire for more information on maternal and fetal health effects. Such misperceptions and knowledge gaps, together with inaccessible care, may help explain increases in prenatal cannabis use during the COVID-19 pandemic (Sokol et al., 2025).

Complementing these findings, Howe et al. (2025) highlighted how stigma may perpetuate misperceptions by shaping where and how pregnant individuals seek information and support. Analyzing posts from an online pregnancy forum, the authors found that pregnant individuals who use cannabis frequently experience stigma within their personal and social networks, including from family and friends (Howe et al., 2025). These stigmatizing experiences were frequently associated with anger and frustration, highlighting the emotional burden of stigma during pregnancy and its potential implications for maternal and fetal health.

The studies by Skelton et al. (2025) and Mian et al. (2025) addressed how clinicians identify, interpret, and respond to cannabis use during pregnancy. Using 2017–2020 Pregnancy Risk Assessment Monitoring System (PRAMS) data from 9 states, Skelton et al. (2025) examined prenatal cannabis screening and counseling practices across states with and without non-medical cannabis legalization. Screening and counseling varied by legalization status, with women in legalization states more likely to be screened for cannabis use and advised against use during pregnancy and lactation (Skelton et al., 2025). Complementing these findings, a mixed-methods study by Mian et al. (2025) examined how mental health clinicians perceive and address prenatal cannabis use (Mian et al., 2025). Clinicians reported that pregnant individuals predominantly use cannabis to manage nausea and morning sickness, appetite, and anxiety. They also described challenges in engaging patients with evidence-based interventions, often due to mixed messaging, low perceived risk, and stigma (Mian et al., 2025).

The expanding body of research on prenatal cannabis use increasingly links exposure to adverse outcomes, including elevated risks of perinatal mortality and neurodevelopmental anomalies which may persist across the lifespan, with evidence of dose–response relationships (Lo et al., 2025). Notably, cannabis use during pregnancy may occur within broader polysubstance use patterns, further complicating risk attribution and interpretation.

Because prenatal cannabis use represents a modifiable risk factor, targeted clinical and public health interventions have the potential to meaningfully reduce pregnancy-related and offspring morbidity and mortality. However, stigma-related barriers to disclosure and support-seeking remain a critical and often underrecognized challenge during pregnancy, potentially limiting the reach and effectiveness of potential interventions.

As evidence continues to evolve regarding the potential harms and benefits of prenatal cannabis use, pregnant individuals must have access to clear, accurate, and evidence-based information. Accordingly, clinicians should serve as trusted sources by offering coordinated, non-stigmatizing conversations grounded in empirical data to support informed decision-making. Ultimately, limited patient–provider communication about cannabis use during prenatal care represents a missed opportunity for early intervention and risk reduction. Given the rising prevalence of prenatal cannabis exposure, further research is urgently needed to clarify its safety and effects on reproductive and offspring health, as well as to ensure that emerging evidence is effectively disseminated to clinicians, pregnant individuals, and public health stakeholders.

## Funding

No financial support was received for the research, authorship, and/or publication of this article.
